# Iatrogenic Obturator Hernia with Ureter as Content: Case Report

**DOI:** 10.15388/Amed.2025.32.1.9

**Published:** 2025-02-18

**Authors:** Alamelu Alagappan, Sambit Tripathy, Biswajit Sahoo, Debabrata Sahani, Manoj Kumar Nayak

**Affiliations:** 1 Department of Radiodiagnosis, All India Institute of Medical Sciences, Bhubaneswar, India E-mail: tuna.manoj@gmail.com; 2 Department of Urology, All India Institute of Medical Sciences, Bhubaneswar, India

**Keywords:** Obturator hernia, ureter, DJ stent, hydroureteronephrosis, obturator foramen, Obturatoriaus išvarža, šlapimtakis, DJ stentas, hidroureteronefrozė, obturatorinė anga

## Abstract

A male in his early 70s presented with complaints of bilateral flank pain for seven months, hematuria for 2 days along with the passage of clots associated with an increased frequency of urination and nocturia. The patient had a history of deep vein thrombosis and was on anticoagulation. On ultrasound, multiple calculi with posterior acoustic shadowing were seen in bilateral kidneys, corroborated by *Computed Tomography* (CT) urography. Laser lithotripsy with DJ stent placement was conducted. The follow-up CT urography showed an inferiorly displaced DJ stent within the right distal ureter and the DJ stent herniating through the right obturator foramen. The right-sided DJ stent was removed after a month. For obturator hernia, the patient is being kept in the follow-up. This case report reviewed the literature and discussed the diagnosis and complications associated with the obturator hernia with the ureter as the content.

## Abstract (it)

Septyniasdešimtmetis vyras septynis mėnesius skundėsi abipusiu šono skausmu, dvi dienas trunkančia hematurija ir krešulių išsiskyrimu, padidėjusiu šlapinimosi dažnumu ir noktiurija. Pacientas sirgo giliųjų venų tromboze ir vartojo antikoaguliantus. Ultragarso tyrimo metu abipus inkstuose buvo matomi daugybiniai akmenys su užpakaliniu akustiniu šešėliu, tai patvirtino kompiuterinės tomografijos (KT) urografija. Atlikta lazerinė litotripsija su DJ stentu. Atlikus vėlesnę KT urografiją, dešiniojo distalinio šlapimtakio viduje buvo pastebėtas į apačią pasislinkęs DJ stentas, ištrūkęs per dešinįjį obturatoriaus plyšį. Po mėnesio dešinės pusės DJ stentas buvo pašalintas. Dėl obturatoriaus išvaržos pacientas toliau stebimas. Šiame atvejo aprašyme apžvelgta literatūra ir aptarta diagnozė bei komplikacijos, susijusios su obturatorine išvarža, kurios turinys – šlapimtakis.

## Introduction

An obturator hernia is an uncommon hernia with high morbidity and mortality. Its incidence is approximately 1%. The small bowel is the content in most cases [1]. The clinical symptoms can be vague and nonspecific. A radiological investigation is essential, with *computed tomography* (CT) playing a diagnostic role in planning surgical intervention. In an extensive literature search, only 4 cases of obturator ureteric hernia have been published so far (as of 20 September 2023, *PubMed, Cochrane*, and *Embase* full-text database search was conducted by using keywords ‘obturator’ ‘ureteric hernia’ ‘ureteral entrapment’ ‘hernia repair’) [2–4]. Herein, we report a case of a right obturator hernia with a ureter as the content following percutaneous nephrolithotomy in a male.

## Case report

**Figure 1:**
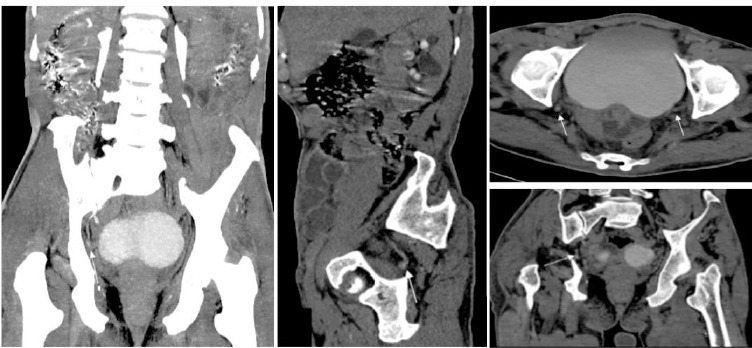
*Computed tomography* (CT) image showing the normal ureteric course in preoperative images

A male in his early 70s presented with complaints of bilateral flank pain for seven months, which was relieved by oral medications. He developed hematuria, 4 to 5 episodes for 2 days, along with the passage of clots associated with an increased frequency and nocturia. He had a history of chronic deep vein thrombosis in his left leg, for which he had been taking the tablet Rivaroxaban 20mg for 2 months before this presentation. On examination, the vitals were stable. The abdomen was soft on palpation without apparent renal angle tenderness. when conducting ultrasound, multiple calculi with posterior acoustic shadowing were seen in bilateral kidneys. On CT urography, two calculi were noted in the right kidney, one in the upper and the other in the lower calyx, with the larger one measuring 12mm. The left kidney showed calculi in the upper and mid calyces, the largest measuring 11x10mm. Bilateral distal ureters showed faint contrast opacification with no evidence of ureteric hernia or calculus. No apparent benign or malignant urological lesions were seen on the nephrogenic phase (Figure 1). Rivaroxaban was stopped, and a hematology consult was taken.

**Figure 2:**
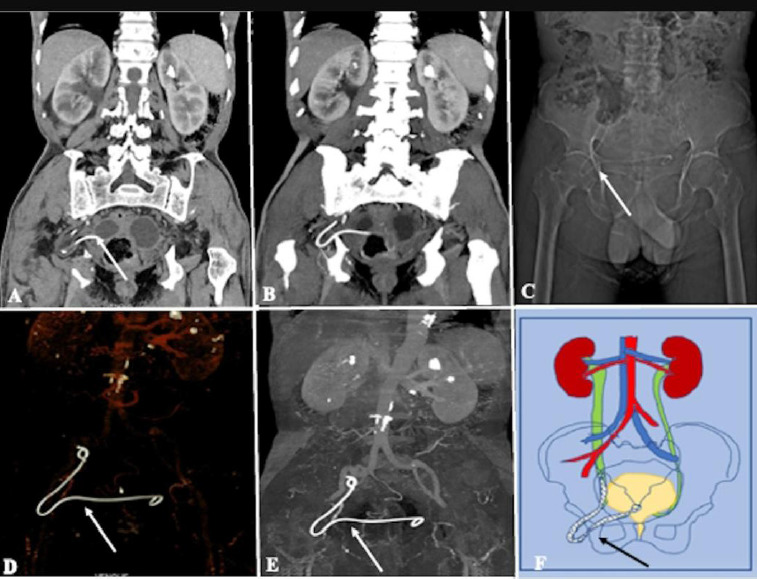
*Computed tomography* (CT) image showing obturator hernia with ureter as content Coronal CT image (a,b) showed a displaced DJ stent within the obturator foramen (long white arrow) with bilateral renal calculi. NCCT scout image (c) and CT reconstructed images (d–e) showed a displaced DJ stent within the obturator foramen (white arrows); schematic illustration (f) of the morphology of the hernia with a DJ stent

No apparent lesion was seen in the cystoscopy. Bilateral retrograde pyelography (RGP) was implemented in a standard manner by using *a Terumo* guidewire, and a ureteric catheter could be advanced without any difficulty, followed by right-sided percutaneous nephrolithotomy under general anesthesia. A C-arm guided infracostal inferior calyceal puncture was done, followed by laser lithotripsy. A small 3mm stone fragment was displaced into the right ureter; hence, the DJ stent was kept *in situ*. No difficulty was felt during the DJ stent insertion, and the procedure was uneventful. Postoperatively, a bilateral leg venous ultrasound was performed, which showed thin webs in the left common femoral vein. No apparent echogenic thrombus was observed. The patient had no flank pain and was discharged. After three weeks, the patient came back for stent removal. The follow-up CT KUB was taken for the patient, showing a displaced right DJ stent with a proximal end in the right mid-ureter and the distal end in the urinary bladder, further confirmed by CT Urography (Figure 2a–e). The right distal ureter and the DJ stent were herniating through the right obturator foramen with a defect measuring approximately 11mm. The schematic illustration of the morphology of the hernia with DJ stent was depicted in the diagram (see Figure 2f). Few calculi were seen in the bilateral kidney, with the largest measuring 11mm in the upper calyx of the left kidney (Figure 2a,b). Left-sided PCNL was performed a month after the right-sided DJ stent had been removed. During the stent removal under local anesthesia, the patient was experiencing a mild discomfort. However, the procedure was uneventful.

The flank pain reduced in the postoperative period, and the patient is being on the follow-up at the time of the case report writing. For obturator hernia, the patient is being kept on the follow-up with the objective to monitor the development of symptoms related to ureteral obstruction or the progression of hydronephrosis on sequential imaging. Regarding DVT, the patient is on the follow-up under hematology.

## Discussion

An obturator hernia is an uncommon variety of abdominal hernias, which is more common in older women; it incurs high morbidity and mortality due to nonspecific symptoms, a delay in diagnosis, and a high probability of bowel strangulation [5]. In our study, the patient was an elderly man. A larger obturator foramen, a wider pelvis, chronic constipation, and COPD can be the predisposing conditions in men.

An obturator hernia contains a bowel, appendix, omentum, or Meckel’s diverticulum. Very rarely, the ureter herniates through the obturator foramen. The possible etiology in our case could be DJ stent-induced traction of the ureter, aided probably by the weakness of the muscles around the obturator foramen. Rodriguez et al., in their study, reported that the right-sided obturator hernia is more common than the left-sided variety because of the sigmoid colon, which overlies the obturator foramen on the left side. It is similar to our case, where our patient also had a right-sided obturator hernia [5]. Clinically, ureteric obturator hernia usually presents with vague and nonspecific symptoms. Specific signs like the Howship Romberg sign (pain along the medial aspect of the thigh due to the compressive effect of the obturator nerve by hernial contents) may be helpful in the diagnosis. Izzo et al., in their case report of ureteric obturator hernia, stated that the patient presented with urinary symptoms due to a compression of the ureter [2]. However, in our case, the patient was asymptomatic and did not have ureteric obstruction or hydroureteronephrosis, which can be attributed to a DJ stent.

CT imaging plays a vital role in diagnosing obturator hernia. In our case, even though the patient had no hydroureteronephrosis, a careful evaluation during CT aided in the diagnosis.

## Conclusion

Ureteric hernia can cause ureteric obstruction, thereby causing hydroureteronephrosis, and, over a longer timeframe, it may impair the renal function, leading to a renal failure. An early diagnosis with a timely surgical intervention is necessary to prevent complications. A higher degree of clinical and radiological suspicion with prior knowledge is indispensable for an early diagnosis and intervention. Computed tomography can play a significant role in the diagnosis as well as when guiding surgical interventions.
